# Examining the Autonomic Nervous System in the Relationship among Heart Rate Variability, Stress Coping, and Cognitive Ability in Individuals with Psychiatric Disorders

**DOI:** 10.3390/jcm11123277

**Published:** 2022-06-08

**Authors:** Melanie Lenger, Nina Dalkner, Karin Schwalsberger, Bianca Hagendorfer, Elena Schönthaler, Alexandra Rieger, Alexander Maget, Frederike T. Fellendorf, Carlo Hamm, Margit Gramer, Alois Hufnagl, Bernd Reininghaus, Eva Z. Reininghaus

**Affiliations:** 1 Department of Psychiatry and Psychotherapeutic Medicine, Medical University of Graz, 8036 Graz, Austria; melanie.lenger@medunigraz.at (M.L.); elena.schoenthaler@medunigraz.at (E.S.); alexander.maget@medunigraz.at (A.M.); frederike.fellendorf@medunigraz.at (F.T.F.); eva.reininghaus@medunigraz.at (E.Z.R.); 2 Therapiezentrum Justuspark, 4540 Bad Hall, Austria; karin.riedrich@gmail.com (K.S.); alexandra.rieger@medunigraz.at (A.R.); carlo.hamm@stud.medunigraz.at (C.H.); alois.hufnagl@bvaeb.at (A.H.); berndreininghaus@web.de (B.R.); 3 Institute for Psychology, Section for Clinical Psychology, Karl-Franzens-Universität Graz, 8010 Graz, Austria; bianca.hagendorfer@gmx.at (B.H.); margit.gramer@uni-graz.at (M.G.)

**Keywords:** depression, heart rate variability, autonomic nervous system, coping strategies, cognitive inhibition

## Abstract

Depression is one of the most severe psychiatric disorders and affects patients on emotional, physical, and cognitive levels. Comorbid somatic conditions, such as cardiovascular diseases, are frequent and affect the quality of life, as well as mortality. Underlying maladaptive autonomic nervous system regulation influences emotional and cognitive processes. This study, thus, aimed to investigate the relationship among heart rate variability (HRV), self-reported coping strategies, executive function, and inhibition in individuals with psychiatric disorders. Data of 97 patients treated in a multi-professional psychiatric rehabilitation center for 6 weeks were analyzed. Subjects underwent psychological tests (Stress Coping Style Questionnaire, Emotional Competence Questionnaire, and Becks Depression Inventory-II), a cognitive test (Color-Word Interference Test), and a 24 h electrocardiogram to record HRV. Patients with higher depression scores had significantly lower HRVs and decreased self-reported abilities for stress coping. Depression severity did not affect cognitive inhibitory abilities. HRV was related to neither coping strategies nor cognitive inhibition abilities. However, lower HRV was related to higher values of Negative Stress Coping (*β* = −0.21, *p* < 0.05). This relationship was fully mediated by depression severity (−4.79, 95% CI: −8.72, −0.72). HRV is not related to quantitative cognitive inhibition, but to the self-reported ability to cope with negative emotions in individuals with psychiatric disorders.

## 1. Introduction

In the last few decades, the research interest in heart rate variability (HRV) and its effect on mental and physical health has increased immensely [1,2,3]. A rigid HRV was associated with mortality in several studies [4,5,6]. Cardiovascular diseases are one of the most common causes of morbidity worldwide [1]. In addition to somatic diseases, psychosocial stress and psychiatric disorders increase the risk for cardiovascular disease [7]. Severe mental illnesses, such as schizophrenia, bipolar disorder, depression, and anxiety disorders are correlated with higher cardiovascular morbidity and partially even higher cardiovascular mortality [7,8,9].

The HRV is the variation in the time interval between consecutive heartbeats in milliseconds reflecting the adaptability of an organism, as it is a proxy for the regulatory ability of the autonomic nervous system [7,10]. The polyvagal theory and the neurovisceral integration model adopt autonomous regulation as an adaptive strategy, which enables the organism to adapt flexibly and is thus involved in the coping of emotions. The polyvagal theory according to Porges [11,12] describes the vagus nerve as being the largest and most important part of the parasympathetic nervous system thus being essentially involved in the parasympathetic regulation of cardiac activity [12]. This theory relates the affective experience, coping with negative emotion, and social behavior to the evolutionary development of the autonomic nervous system. Appelhans and Luecken [10] examined the main assumptions of the polyvagal theory concerning the association between heart rate variability and the ability to cope with negative emotions. Accordingly, a higher HRV supports emotional self-regulation and a reduced HRV indicates emotional dysregulation [13]. Another model for the connection between physiological processes and emotion regulation is the neurovisceral integration model according to Thayer and Lane [14]. They assume that cortical and subcortical structures influence cardiovascular, emotional, and behavioral processes. It is known that patients with depression often have maladaptive emotion regulation strategies [15].

Compared to healthy individuals, patients with depression have a significantly lower overall HRV as well as a reduced parasympathetic activity, which is important for the regeneration process [3,8,16,17]. Among others, the relationship between depression and cardiovascular diseases can be explained by the functioning of the autonomic nervous system. HRV depicts the interplay between the sympathetic and parasympathetic nervous systems and is an important marker for the ability to cope with negative emotions [10].

Additionally, depression is associated with deficits in several cognitive functions [18]. This makes information processing and the flexibility of attention more difficult [15]. Inhibitory abilities seem to play a crucial role in affective disorders because of the maladaptive coping of emotions [15,18,19]. Previous studies assumed that the expression of HRV may be positively related to cognitive abilities such as executive function [20,21,22,23]. The executive core area of inhibition was of particular interest here, as it is also involved in emotion control. The relationship between regulatory functions and HRV is already well known in the literature [20]. Several theories describe the common neurobiological basis for these functions as a possible explanation for the relationship [21]. A recent study also demonstrates that training in executive function improves HRV and vice versa [3].

Nevertheless, there is no evidence for the relationship among executive function, HRV, and the subjective assessment of regulatory functions and coping. Therefore, the current study deals with the relationship between the expression of HRV and (1) coping, as well as (2) executive function in individuals with psychiatric disorders and symptoms of depression. We assume that psychiatric patients with symptoms of depression show maladaptive parameters of HRV, which can be linked to clinical symptoms, self-reported coping strategies, emotion- and stress regulation, and cognitive inhibition. Hence, the aim of the study was to investigate the association of different HRV parameters (24 h, day and night) and the self-reported ability to cope with negative emotions as well as the objectively measured ability to regulate and inhibit cognitive processes, and if this association is influence by depressive symptoms.

## 2. Materials and Methods

### 2.1. Sample

The data were collected within a large-scale pilot study on the neurobiological background of burnout syndrome disorders, which was conducted in 2015 at an Austrian psychiatric rehabilitation clinic. This rehabilitation clinic provides a psychiatric rehabilitation program with a duration of 6 weeks, consisting of a thorough examination at the time of admission and discharge (medical examination, blood count analysis, and psychological diagnostics) and intensive therapeutic interventions: psychotherapeutic sessions, group sessions, ergotherapy, physiotherapy and sport, healthy diet, and dietary consultation. Each patient undergoing the rehabilitation program was included in the study if meeting the criteria. The following inclusion criteria were defined: diagnosed depressive disorder (F3) neurotic, stress-related and somatoform disorders (F4), and other psychiatric patients with depressive symptoms. Voluntary participation, signed informed consent, and a psychiatric rehabilitation stay for at least 3 weeks were obligatory. Patients were excluded if they refused to participate, were diagnosed with an addiction syndrome, had moderate or severe intellectual impairments, or suffered from a condition that impaired their cognitive performance, for example, an active severe organic brain disease or a form of dementia.

The present study was approved by the Ethics Committee of the Federal State Upper Austria by the current revision of the Declaration of Helsinki, the ICH Guideline for Good Clinical Practice, and the applicable regulations (EK number: E-24-14). All participants signed informed consent.

### 2.2. Study Design

At the beginning of the rehabilitation treatment, the patients’ medication was recorded and the medical, and psychiatric diagnoses were documented within a clinical interview by psychiatrists (for an overview see Table 1). Concerning the psychological diagnostic process, psychological inventories were conducted within the first 3 days of admission. To record the severity of depressive symptoms, the German version of the Becks Depression Inventory-II (BDI-II) by Hautzinger, Keller, and Kühner [24] was used for self-assessment. To record the coping with emotions, the subscale “Regulation and control of one’s feelings” of the Emotional Competence Questionnaire (EKF) by Rindermann [25] was used. The Color-Word Interference Test (FWIT) according to Stroop [26] was used to determine the executive function inhibition.

#### Additional Exploratory Analysis

Data of the Stress Coping Style Questionnaire (German: Stressverarbeitungsfragebogen 78: short SVF 78), which assesses the usage of Negative Stress Coping strategies such as rumination and self-incrimination, were included in the statistical analysis. Its additional value in answering the hypothesis was revealed during the analytical process [27].

### 2.3. Psychophysiological Methods

A 24 h HRV measurement was performed once within the first week following admission to get an overall impression of the adaptability of the organism. After the trained nursing staff had properly placed the electrocardiogram (ECG), the patients wore the device for 24 h, while the remainder of the day-to-day therapy continued as usual. To quantify the HRV, the standard deviation of all RR intervals (SDNN) and the square root of the mean value of the sum of the squared differences between adjacent RR intervals (RMSSD) were used and calculated. The mean value during the day (6 a.m. to 9 p.m.), night (10 p.m. to 5 a.m.), and the entire 24 h was calculated for both HRV parameters. The timing was chosen on the basis of a night’s rest at the rehabilitation center.

Furthermore, the day–night changes in the used HRV parameters were analyzed, since the expression of the HRV in healthy individuals is influenced by circadian rhythms and shows significant fluctuations over 24 h. In healthy people, the mentioned HRV parameters increase during the night and reach their maximum values in the last deep sleep phase. During the day, the HRV values drop again and reach their lowest value in the late afternoon [17].

### 2.4. Statistical Analyses

The statistical analyses to answer the hypotheses were carried out with the German version of SPSS 26 (https://www.ibm.com/support/pages/downloading-ibm-spss-statistics-26, accessed on 1 March 2022). The alpha level of the analyses was set at *p* = 0.05 (two-tailed). In order to conduct the calculations, all variables used were checked visually and statistically for outliers. Age and sex correlated significantly with nighttime SDNN (*r* = −9.311; *p* < 0.001). Therefore, according to the results of the literature, age, sex and medication were defined as control variables. To test the association between self-reported coping of emotion and HRV parameters, a hierarchical regression analysis was performed. The variables influencing HRV, i.e., age, sex, body mass index, and drugs, were defined as control variables since they significantly correlated with the severity of depression.

To test the relationship between the severity of depressive symptoms and the HRV-parameters, a simple linear regression with the HRV parameters SDNN, RMSSD, SDNN24 h, and RMSSD24 h was calculated.

Since the raw values of the SDNN and RMSSD were not normally distributed but showed a right-skewed distribution, a logarithm transformation was performed for all HRV parameters for further analyses.

## 3. Results

### 3.1. Sample Description

The data of 697 patients were analyzed. After the selection process (see Section 2), a total of 97 patients between the ages of 22 and 73 years were included in the final sample, including 39 men and 58 women (average age M = 53.55 years; SD = 7.98), with an average BDI-II score of 19.67 (SD = 10.34). For an overview see Table 2.

### 3.2. Depression and HRV

For an overview of the psychophysiological data, please see Table 3. Results show that the severity of depression, measured with the BDI-II, was a statistically significant predictor for the SDNN daily value (*t*_86_ = −2.9, *p* < 0.01), the 24 h SDNN value (*t*_89_ = −2.41, *p* < 0.05), and the 24-h RMSSD value (*t*_86_ = −2.02, *p* < 0.05).

### 3.3. Depression and Ability to Cope with Emotions

The assumed negative correlation between the severity of self-assessed depression and the ability to cope with emotions was determined using hierarchical regression. The first model included the control variable medication and was significant (F_1.95_ = 9; *p* < 0.05; *R* = 0.29; *R*^2^ = 0.08). The control variable medication predicted 8.7% of the variance in the ability to cope with negative emotions. Furthermore, adding severity of the depression (BDI-II) in a second step led to a significant model (F_1,94_ = 13.4; *p* < 0.001; *R* = 0.45; *R*^2^ = 0.18). This increased the explanation of variance by 11.4% to a total of 20.1%. Medication (t_89_ = 2.98, *p* < 0.05) and the BDI-II (t_89_ = −3.66, *p* < 0.001) were statistically significant predictors for the extent of emotional coping measured with the EKF. The regression model showed that, if the severity of the depression increases by one unit, according to the BDI-II, the ability for emotional coping decreases by 0.73 units.

### 3.4. Depression and Cognitive Inhibition

There was no statistically significant correlation between the severity of depressive symptoms and the FWIT performance. It should be noted that all patients had an average or above-average test performance according to *T*-values in the FWIT manual (range *T*-values = 40 to 72).

### 3.5. HRV and Emotional Coping Strategies/Inhibition

Likewise, the assumed relationship between the ability to cope with emotions and the expression of the HRV parameters, as well as between the FWIT performance and the HRV parameters, was not statistically significant (*p* > 0.05).

### 3.6. Day–Night Change in HRV Parameters

For the change analysis of the 24 h HRV parameters, paired *t*-tests were carried out to compare the mean values. The comparison of the SDNN values day and night did not show any statistically significant difference in the mean values. As a result, the patients’ measured nighttime SDNN values did not differ from the daytime SDNN values. The mean values of the RMSSD of the day differed significantly from the RMSSD mean values of the night, with a small effect (*d* = 0.22). At night, the RMSDD mean values were significantly higher than during the day (*t*_91_ = −2.07, *p* < 0.05).

In a mediation analysis, the SVF 78 subscale “Negative Stress Coping” was included to consider a further facet of emotion regulation, since the EKF’s validity as an instrument to measure coping of emotions in depressed patients was questioned in similar studies. The additional analysis showed that a lower level of daily SDNN values was related to higher usage of Negative Stress Coping (*β* = −0.21, *p* < 0.05), and this relationship was fully mediated by the severity of depression (indirect effect; −4.79, 95% CI: −8.72, −0.72).

## 4. Discussion

The present work aimed to investigate the relationship between HRV and both emotional and stress coping strategies as well as executive functions in individuals with psychiatric disorders and depressive symptoms. Patients with relevantly elevated depression scores had significantly lower HRV and improper self-reported abilities to cope with negative emotions and stress. However, expression severity did not relate to cognitive inhibitory abilities. In patients undergoing a psychiatric rehabilitation, HRV was related neither to the ability to cope with emotions nor to the cognitive inhibition ability. Additionally, a lower HRV was related to higher values for the subscale of self-reported “Negative Stress Coping”. This relationship is fully mediated by depression severity. Interestingly, the HRV was not related to cognitive inhibition, but self-reported regulation abilities in patients with psychiatric disorders.

Numerous studies have reported that depression is associated with lower HRV, compared to mentally healthy individuals [2,3,8,16,17,28]. Accordingly, it is expected that people with a higher score in self-reported depression would have less variability in their heart rate [8,17]. Depression affects patients on different levels, the most obvious being the emotional level. Anhedonia, lack of motivation, and low self-worth are just some of the characteristics of depression. In addition to the emotional level, cognitive performance is also reduced in patients with depression. This is expressed by brooding, rumination, predominantly negative thoughts, concentration difficulties, and memory deficits [15,29,30].

On the basis of this aspect of depression, recent studies assumed that the severity of depression was negatively related to the ability to cope with one own’s emotions [15]. A uniform correlation was expected concerning the executive function of cognitive inhibition. According to the polyvagal theory according to Porges [11,12] and the neurovisceral integration model according to Thayer and Lane [14], a positive relationship was assumed between the regulation of the autonomic nervous system, which was mapped using the HRV and the ability to cope emotionally.

As expected, our study showed that patients with depressive symptoms in rehabilitation had lower levels of SDNN daytime values, SDNN over 24 hours, and RMSSD values over 24 hours. Regarding the severity of depression, this result indicates that the total variability over the day and the entire period of the measurement over 24 hours was lower in people with higher values in the BDI-II than in people who had lower values in the BDI-II. It is important to note that the SDNN is activity-dependent [7]. This means that the SDNN increases with greater physical activity. Since patients did not keep an activity diary during the 24-hour HRV measurement, the interpretation of the daily value of the SDNN must be viewed critically. During the treatment phase, patients took part in the rehabilitation program as usual and also received physiotherapy, indicating increased physical activity.

Consideration of sleep quality could clarify why there was no connection between the severity of depression and the HRV nighttime values. The relationship between HRV and sleep quality is characterized by the fact that people without sleep problems have higher variability in heart rate at night than people with sleeping disorders. According to the results of Burton and colleagues [31], a low HRV predicts poor sleep quality in people with sleep disorders. In contrast to a healthy control group, RMSSD was significantly reduced in people with sleep disorders in their study. Further studies could include sleep quality as a control variable. In the present study, a statistically significant relationship between the severity of depression and HRV was confirmed; this goes in line with the results of Udupa and colleagues [32].

Aligned with the results of Hartmann and colleagues [30], no general relationship with all HRV parameters in psychiatric patients with depressive symptoms could be confirmed in the current study for age or sex. A possible explanation for this could be the already reduced HRV in patients with major depression.

Depression severity was also related to the ability to cope with one own´s emotion. The results showed that patients with a higher degree of depression severity (according to BDI-II) had lower values in the EKF basic scale “Regulation and control of one’s own feelings”. This result agrees with that of Joorman and Vanderlind [15]. The authors explained that patients with depression are more likely to use inappropriate strategies to cope with emotions and have problems using effective adaptation strategies. Joormann and Gotlib [33] also established maladaptive strategies of emotional coping in patients with severe depressive episodes.

According to the results of this study, the severity of depression is not related to the interference performance according to the FWIT test. Previous studies came to inconsistent results. For example, Kertzman et al. [34] found no correlation between the processing time of the interference task in patients with major depression. Gohier and colleagues [19], on the other hand, concluded that people with major depression solved the interference task of the FWIT test significantly more slowly than healthy people. However, in the present study, no comparison was made with a healthy control group. It should also be noted that the patients’ interference performance was not below average.

Concerning the relationship between HRV and Negative Stress Coping strategies (measured by the SVF), a negative correlation was assumed on the basis of the current literature. The analysis of this hypothesis surprisingly showed that the used HRV parameters SDNN and RMSSD were not related to the ability to cope with emotions, recorded with the EKF scale “Regulation and control of one’s own feelings”. Holzman and Bridgett [35] reported in their meta-analytical review that only two strategies of emotional coping, namely, suppression and distraction, show a connection with the expression of HRV. People who frequently use the strategies mentioned have lower variability in heart rate. In comparison to the present study, the question arises whether the EKF subscale “Regulation and control of one’s own feelings” is comparable with the strategies mentioned in the two studies described. In terms of content, this should be viewed more critically, as the above-mentioned EKF subscale rather deals with the emotions of anger and rage. The strategies for coping with stress, which were evaluated in a subsequent additional analysis, would be better comparable. The scale for the usage of Negative Stress Coping strategies of the SVF was subsequently compared with HRV parameters and showed a significant correlation, which was mediated by the severity of depression.

Nevertheless, this study failed to find a statistically significant relationship between HRV parameters and the FWIT performance. Contrary to the initial assumption, the level of HRV was not related to the inhibitory abilities of the patients examined. Gathright and colleagues [36] examined the association between executive function and HRV regarding depressive symptoms, albeit in a sample of patients with heart failure. Furthermore, they analyzed different HRV indicators (high frequencies) compared to other studies. In line with the present study, the authors found that BDI-II values were related to HRV, but not to executive functions or inhibitory ability.

The HRV parameters were not associated with the executive function test, FWIT. A possible explanation for this finding could be the fact that none of the examined individuals had a below-average executive performance. Furthermore, it should be noted that subjects were in psychiatric rehabilitation. It can, therefore, be assumed that the patients who took part in the study did not suffer from severe depressive symptoms that required inpatient treatment. In some cases, according to the self-assessment measured with BDI-II, the depressive symptoms of the patients were not severe.

### Limitations

Since there was no control group, the results and interpretation are limited to psychiatric patients. Since we investigated all patients undergoing a psychiatric rehabilitation program, patients with different diagnoses, namely unipolar, bipolar, and anxiety disorders, are included. This influenced the study outcome. However, these mental illnesses have very high comorbidity, therefore the study outcome is still valuable for this patient group.

Furthermore, there was a high variance of medication, and the effects of different substances were not considered. Nevertheless, we obtained, significant results and the explained variance was higher for the dependent variables of the present analyses than for the medication. Furthermore, the intensity of physiotherapy and sports programs varied according to patients, which could have influenced the study outcome.

The HRV measurement also had its limitations. The measurements were not recorded on the same day for all patients, and the BDI, EKF, SVF, and cognitive tests were not measured on the same day, because the admission process of the rehabilitation program did not allow it. However, the measurements took place within the first 3 days, where the therapeutic effect is minor. Ideally, a second HRV measurement at the end of the rehabilitation program would have been ideal but was not possible due to a lack of resources. 

Additionally, individuals with psychiatric disorders showed no clinically relevant deficits in executive function in the present study. Since the majority of patients did not suffer from acute episodes during the rehabilitation stay, further investigation is needed for acute episodes.

## 5. Conclusions

The present study confirms the assumption that individuals with a higher degree of depression have a lower HRV in the parameters SDNNday, SDNN24h, and RMSSD24h. The severity of depression, therefore, influenced the regulation of the autonomic nervous system, which also affects the control and regulation of one own’s feelings. However, the severity of depression did not affect executive functions. Accordingly, the assumption that there is a connection between HRV parameters and the ability to cope with negative emotions could not be confirmed. In any case, further studies are needed regarding the relationship between HRV and emotional and cognitive skills. The result of the additional analysis, which showed that a lower HRV is related to higher usage of Negative Stress Coping strategies and that this relationship is fully mediated by the severity of depressive symptoms, was significant. Interestingly, in psychiatric patients with depressive symptoms, the physiological level of HRV was not related to the recorded executive function ability but to the self-assessment of the coping strategies. This may indicate that symptoms of depression are more likely to affect emotional skills and self-perception than cognitive skills such as executive functions. This aspect and the existing relationship between the severity of depression and the HRV indicate the benefits of including HRV training, such as biofeedback-supported breathing training [37], in the clinical intervention of mental disorders to restore the autonomous balance of patients with depressive symptoms.

## Figures and Tables

**Table 1:** Overview of obtained parameters.

	Abbreviation	Parameters
**Sociodemographic data**	-	Date of birth
	-	Sex
**Physical examination**	Meds	Current psychiatric medication
	HRV	Heart rate variability SDNN *, RMSSD **
**Psychological** **Inventories**	BDI-II	Becks Depression Scale II *Assesses the severity of depressive symptoms* via *self-report*
	SVF-78	SVF-78—Stress Coping Questionnaire *Subcale: “Negative Stress Coping”*
	EKF	Emotional Competence Questionnaire *Subscale: “regulation and* *control of one’s own feelings”* *assesses the ability* to cope with one own’s *emotions* via *self-report*
	FWIT	Color-Word Interference Test *Using the color-word* *incongruence principle of J.R. Stroop, the FWIT measures* *concentration and attention as well as the ability to inhibit* *executive functions*

Note: * SDNN: the standard deviation of all RR intervals. ** RMSSD: the square root of the mean value of the sum of the squared differences between adjacent RR intervals.

**Table 2:** Overview of sociodemographic and clinical data.

Sample Description			
Sex	Female	*N* = 58	59.8%
	Male	*N* = 39	40.2%
Age in years		M = 53.55	SD = 7.98
BDI-II		M = 19.67	SD = 10.34

M = mean, SD = standard deviation, BDI-II = Becks Depression Inventory

**Table 3:** Overview of the HRV parameters.

	*N*	M	SD	Min	Max
SDNN day	97	50.99	15.05	20.82	109.98
SDNN night	97	53.92	23.37	20.05	142.65
SDNN 24 h	97	51.97	16.53	23.00	120.87
RMSSD day	92	19.91	10.40	4.03	49.75
RMSSD night	92	21.23	11.48	3.98	58.13

Note: SDNN = standard deviation of all RR intervals; RMSSD = sum of the squared differences between adjacent RR intervals.

## Data Availability

The data presented in this study are available on request from the corresponding author, due to ongoing analysis.

## References

[1] Wnent J., Gräsner J.T., Maurer H. (2020). Out-of-Hospital Cardiopulmonary Resuscitation. Anasthesiol. Intensivmed. Notfallmed. Schmerzther. AINS.

[2] Caldwell Y.T., Steffen P.R. (2018). Adding HRV biofeedback to psychotherapy increases heart rate variability and improves the treatment of major depressive disorder. Int. J. Psychophysiol..

[3] Pinter A., Szatmari S., Horvath T., Penzlin A.I., Barlinn K., Siepmann M., Siepmann T. (2019). Cardiac dysautonomia in depression—Heart rate variability biofeedback as a potential add-on therapy. Neuropsychiatr. Dis. Treat..

[4] Wulsin L.R., Horn P.S., Perry J.L., Massaro J.M., D’Agostino R.B. (2015). Autonomic Imbalance as a Predictor of Metabolic Risks, Cardiovascular Disease, Diabetes, and Mortality. J. Clin. Endocrinol. Metab..

[5] Villareal R.P., Liu B.C., Massumi A. (2002). Heart rate variability and cardiovascular mortality. Curr. Atheroscler. Rep..

[6] Pei J., Tang W., Li L.-X., Su C.-Y., Wang T. (2015). Heart rate variability predicts mortality in peritoneal dialysis patients. Ren. Fail..

[7] Lohninger A. (2017). Herzratenvariabilität: Das HRV-Praxis-Lehrbuch.

[8] Koch C., Wilhelm M., Salzmann S., Rief W., Euteneuer F. (2019). A meta-analysis of heart rate variability in major depression. Psychol. Med..

[9] Perna G., Riva A., Defillo A., Sangiorgio E., Nobile M., Caldirola D. (2020). Heart Rate Variability: Can It Serve as a Marker of Mental Health Resilience?: Special Section on “Translational and Neuroscience Studies in Affective Disorders” Section Editor, Maria Nobile MD, PhD. J. Affect. Disord..

[10] Appelhans B.M., Luecken L.J. (2006). Heart Rate Variability as an Index of Regulated Emotional Responding. Rev. Gen. Psychol..

[11] Porges S.W. (1995). Orienting in a Defensive World: Mammalian Modifications of Our Evolutionary Heritage. A Polyvagal Theory. Psychophysiology.

[12] Porges S.W. (2009). The polyvagal theory: New insights into adaptive reactions of the autonomic nervous system. Clevel. Clin. J. Med..

[13] Aldao A., Nolen-Hoeksema S., Schweizer S. (2010). Emotion-regulation strategies across psychopathology: A meta-analytic review. Clin. Psychol. Rev..

[14] Thayer J.F., Lane R.D. (2000). A model of neurovisceral integration in emotion regulation and dysregulation. J. Affect. Disord..

[15] Joormann J., Vanderlind W.M. (2014). Emotion Regulation in Depression: The role of biased cognition and reduced cognitive control. Clin. Psychol. Sci..

[16] Lesnewich L.M., Conway F.N., Buckman J.F., Brush C.J., Ehmann P.J., Eddie D., Bates M.E. (2019). Associations of depression severity with heart rate and heart rate variability in young adults across normative and clinical populations. Int. J. Psychophysiol..

[17] Stapelberg N.J., Hamilton-Craig I., Neumann D.L., Shum D.H., McConnell H. (2012). Mind and heart: Heart rate variability in major depressive disorder and coronary heart disease—A review and recommendations. Aust. N. Z. J. Psychiatry.

[18] Schmid M., Hammar Å. (2013). Cognitive function in first episode major depressive disorder: Poor inhibition and semantic fluency performance. Cogn. Neuropsychiatry.

[19] Gohier B., Ferracci L., Surguladze S.A., Lawrence E., El Hage W., Kefi M.Z., Allain P., Garre J.B., Le Gall D. (2009). Cognitive inhibition and working memory in unipolar depression. J. Affect. Disord..

[20] Thayer J.F., Hansen A.L., Saus-Rose E., Johnsen B.H. (2009). Heart Rate Variability, Prefrontal Neural Function, and Cognitive Performance: The Neurovisceral Integration Perspective on Self-regulation, Adaptation, and Health. Ann. Behav. Med..

[21] Thayer J.F., Lane R.D. (2009). Claude Bernard and the heart–brain connection: Further elaboration of a model of neurovisceral integration. Neurosci. Biobehav. Rev..

[22] Al Hazzouri A.Z., Elfassy T., Carnethon M.R., Lloyd-Jones D.M., Yaffe K. (2017). Heart Rate Variability and Cognitive Function in Middle-Age Adults: The Coronary Artery Risk Development in Young Adults. Am. J. Hypertens..

[23] Colzato L.S., Jongkees B.J., De Wit M., Van Der Molen M.J., Steenbergen L. (2018). Variable heart rate and a flexible mind: Higher resting-state heart rate variability predicts better task-switching. Cogn. Affect. Behav. Neurosci..

[24] Hautzinger M., Keller F., Kühner C. (2009). BDI-II. Beck Depressions-Inventar (2. Aufl.). Manual.

[25] Rindermann H. (2009). Emotionale-Kompetenz-Fragebogen: EKF.

[26] Bäumler G. (1985). Farbe-Wort-Interferenztest Nach JR Stroop.

[27] Janke W., Erdmann G. (2002). SVF 78. Eine Kurzform des Stressverarbeitungsfragebogens SVF 120. Manual.

[28] Siepmann M., Aykac V., Unterdörfer J., Petrowski K., Mueck-Weymann M. (2008). A Pilot Study on the Effects of Heart Rate Variability Biofeedback in Patients with Depression and in Healthy Subjects. Appl. Psychophysiol. Biofeedback.

[29] Gauggel S., Lautenbacher S. (2010). Neuropsychologie Psychischer Störungen.

[30] Hartmann R., Schmidt F.M., Sander C., Hegerl U. (2019). Heart Rate Variability as Indicator of Clinical State in Depression. Front. Psychiatry.

[31] Burton A.R., Rahman K., Kadota Y., Lloyd A., Vollmer-Conna U. (2010). Reduced heart rate variability predicts poor sleep quality in a case–control study of chronic fatigue syndrome. Exp. Brain Res..

[32] Udupa K., Sathyaprabha T.N., Thirthalli J., Kishore K.R., Lavekar G.S., Raju T.R., Gangadhar B.N. (2007). Alteration of cardiac autonomic functions in patients with major depression: A study using heart rate variability measures. J. Affect. Disord..

[33] Joormann J., Gotlib I.H. (2010). Emotion Regulation in Depression: Relation to Cognitive Inhibition. Cogn. Emot..

[34] Kertzman S., Reznik I., Hornik-Lurie T., Weizman A., Kotler M., Amital D. (2010). Stroop performance in major depression: Selective attention impairment or psychomotor slowness?. J. Affect. Disord..

[35] Holzman J.B., Bridgett D.J. (2017). Heart rate variability indices as bio-markers of top-down self-regulatory mechanisms: A meta-analytic review. Neurosci. Biobehav. Rev..

[36] Gathright E.C., Walter F.A., Hawkins M.A., Spitznagel M.B., Hughes J.W., Gunstad J. (2016). Executive function moderates the relationship between depressive symptoms and resting heart rate variability in heart failure. J. Behav. Med..

[37] Lehrer P.M., Gevirtz R. (2014). Heart rate variability biofeedback: How and why does it work?. Front. Psychol..

